# Health-Related Quality of Life and Patient Experience in Oncology Palliative Care Within the Saudi Model of Care Framework: Evidence from the Qassim Health Cluster, Saudi Arabia

**DOI:** 10.3390/healthcare13222936

**Published:** 2025-11-17

**Authors:** Fatmah Alribdi, Musaad Aljaloud, Abdullah Alqwaee, Abdulsalam Alfawzan, Annalyn Camba, Abdulrahman Al Mesned, Mohammed Awad Alanazi, Ahmed Almeman, Khuzama Alkhalaf, Albandary Freeh Alanazi, Musa Mohammed Alharbi

**Affiliations:** 1 Saudi Model of Care Department, Qassim Health Cluster, Buraidah 52384, Saudi Arabia; musaad.jaloud@gmail.com (M.A.); abdulsalamalfawzan@gmail.com (A.A.); cambaannalyn0@gmail.com (A.C.); malsumairy@gmail.com (M.A.A.); kh-1996@outlook.sa (K.A.); albandrya@moh.gov.sa (A.F.A.); 2 HealthCare Delivery Department, Qassim Health Cluster, Buraidah 52367, Saudi Arabia; dr_abdullahh@yahoo.com (A.A.); mmalharbi1@moh.gov.sa (M.M.A.); 3 Department of Cardiology, Prince Sultan Cardiac Center, Buraidah 52366, Saudi Arabia; almesnid@yahoo.com; 4 Department of Internal Medicine, College of Medicine, Qassim University, Buraidah 51452, Saudi Arabia; meman@qu.edu.sa

**Keywords:** palliative care, oncology, quality of life, patient experience, Qassim, Saudi Arabia

## Abstract

**Background**: This study was conducted within the Qassim Health Cluster as part of efforts to operationalize the Saudi Model of Care (SMoC). It aims to evaluate the outcomes and value of palliative care services in alignment with SMoC by applying patient-centered measurement tools. In Saudi Arabia, Vision 2030 has spurred significant expansion in palliative care, yet challenges persist in home-based care and consistent outcome measurement. Current Quality-Adjusted Life Year (QALY) methodologies often fail to capture patient-centered dimensions (emotional, spiritual, and social support), leading to gaps in outcome assessments. **Methods**: This cross-sectional descriptive study characterized 147 oncology palliative care patients in Qassim, Saudi Arabia, from January to December 2024. Data on demographics, diagnoses, care duration, health-related quality of life (EQ-5D-5L), and patient experiences were collected via face-to-face interviews. Although QALY was conceptually referenced, the study utilized EQ-5D-5L as a proxy measure for patient-perceived health status. **Results**: The cohort was predominantly older females (64.63%) with various cancer diagnoses, primarily breast cancer (29.25%), and a long duration of palliative care. Patients reported significant impairments in mobility (21.77%), self-care, daily activities, and a high prevalence of pain and anxiety/depression. However, most felt respected by their care team (85.71%), experienced effective symptom management (68.03%), and were consistently involved in decision-making (68.03%). **Conclusions**: This study provides baseline data on oncology palliative care in Qassim, highlighting complex patient needs alongside positive perceptions of care. QALY methodologies must be refined to better capture patient-centered benefits and inform resource allocation, contributing to more responsive and effective palliative care services. However, due to the cross-sectional design, causal relationships between care exposure and outcomes cannot be inferred; hence, the findings should be interpreted as descriptive rather than causal.

## 1. Introduction

Palliative care, as defined by the World Health Organization (WHO), is an interdisciplinary approach aimed at enhancing the quality of life of patients and their families confronting life-limiting illnesses through early identification, accurate assessment, and comprehensive treatment of physical, psychosocial, and spiritual suffering [1]. The global burden of chronic and noncommunicable diseases highlights the critical need for robust palliative care services. Each year, an estimated 56.8 million people, including 25.7 million in the last year of life, require palliative care [2]. Palliative care is required for a wide range of diseases, with the majority of adults in need of palliative care for chronic conditions, such as cardiovascular diseases (38.5%), cancer (34%), chronic respiratory diseases (10.3%), AIDS (5.7%), and diabetes (4.6%) [2]. These and other conditions, such as advanced cancers, heart failure, chronic obstructive pulmonary disease, and neurological disorders, contribute significantly to morbidity and mortality worldwide, necessitating comprehensive care beyond curative treatments to address holistic patient needs. This imperative is further highlighted by projections indicating that, by 2060, an estimated 48 million people, representing 47% of all global deaths, will experience serious health-related suffering, an 87% increase from the 26 million in 2016 [3]. Despite its recognized importance and escalating demand, access to and provision of quality palliative care remain significant global challenges, and only about 14% of people who need palliative care currently receive it. Currently, 45% of countries lack access to palliative care, with 83% of future palliative care needs projected to occur in low- and middle-income countries [4].

The global demographic shift towards an aging population, coupled with advancements in medical science that prolong life in chronic conditions, has amplified the demand for palliative care [5]. This growing need emphasizes the urgency for healthcare systems to integrate palliative care principles across all levels of care, from primary health services to specialized tertiary centers. In line with these efforts, this study aimed to evaluate the outcomes and value of palliative care services under the Saudi Model of Care (SMoC), using patient-centered measurement tools to guide value-based service development. Effective palliative care not only alleviates physical symptoms but also supports psychological well-being, social connections, and spiritual peace, thereby enhancing the overall human experience of patients and providing crucial support for their caregivers. Understanding the specific needs and experiences of palliative care oncology populations is fundamental for developing and refining patient-centered and culturally appropriate services.

Health-related quality of life (HRQoL), a critical outcome measure in palliative care, encompasses an individual’s subjective perception of the impact of an illness and its treatment on their physical, emotional, social, and functional well-being [6]. Tools such as the EuroQol Five-Dimension Five-Level Questionnaire (EQ-5D-5L) are widely used to assess HRQoL, providing a standardized framework to capture patients’ self-reported health status across key dimensions, such as mobility, self-care, usual activities, pain/discomfort, and anxiety/depression [7]. The collective assessment of HRQoL in a patient population offers invaluable insights into the burden of illness and the effectiveness of care interventions from the patient’s perspective, guiding clinical practice and policy development. Beyond objective measures, the subjective experience of care, including perceptions of respect, effective symptom management, and involvement in decision-making, significantly influences a patient’s overall satisfaction and quality of life. These aspects reflect the extent to which care is patient-centered and aligned with individual values and preferences.

In Saudi Arabia, efforts to expand palliative care services have grown significantly in line with the Vision 2030 healthcare reforms [8]. Historically, palliative care services in the Kingdom have been nascent, often integrated within oncology departments, or provided informally by family members [9]. However, in line with the Vision 2030 healthcare transformation agenda, there has been a coordinated effort to expand and formalize palliative care provision. These include initiatives to establish dedicated palliative care units, enhance professional training, and increase public awareness [10]. Despite these advancements, the provision of comprehensive palliative care across the vast geographical expanse of Saudi Arabia remains uneven, and there is a paucity of published research detailing the characteristics, quality of life, and experiences of palliative care patients in oncology within specific regions. Understanding the unique challenges and opportunities within the Saudi Arabian context, including the cultural sensitivities surrounding end-of-life discussions and family involvement, is crucial for effective service delivery.

The Qassim region, located in central Saudi Arabia, represents a significant and distinct demographic area within the Kingdom. As healthcare services continue to evolve and decentralize, localized data from regions such as Qassim have become increasingly vital for informing regional healthcare planning and resource allocation. Qassim is currently underrepresented in the national palliative care literature, reflecting a broader research gap in regional-level data [11]. Most existing studies tend to focus on centralized urban regions such as Riyadh and Jeddah, with Qassim often grouped under more general “central region” analyses. While general trends in palliative care development may apply nationally, specific patient profiles, prevalent diagnoses, and patient experiences can vary considerably between regions because of differences in healthcare infrastructure, cultural practices, and community awareness [12]. Therefore, there is a clear need for region-specific studies to comprehensively understand patient experiences, service accessibility, and cultural factors that influence care delivery in areas such as Qassim. A detailed understanding of the oncology palliative care population in Qassim is thus essential for tailoring services that are responsive to local needs and contributing to the broader national picture of palliative care development.

Despite the increasing focus on palliative care in Saudi Arabia, a notable gap remains in the literature regarding comprehensive descriptive studies that characterize oncology palliative care patient populations, their health-related quality of life, and their perceptions of care within specific regional contexts [13]. Such foundational data are critical for benchmarking current service provisions, identifying areas of unmet needs, and guiding future interventions aimed at improving patient outcomes. Without a clear understanding of who these patients are, their primary concerns, and how they perceive the care they receive, developing evidence-based strategies to enhance palliative care services is challenging.

The conceptual framework of this study was guided by value-based healthcare principles, particularly the Quality-Adjusted Life Year (QALY) model. Although direct QALY calculations were not performed, the use of the EuroQol Five-Dimension Five-Level Questionnaire (EQ-5D-5L) enables future utility-based outcome assessments in alignment with the SMoC. To the best of our knowledge, this is the first study to report on oncology palliative care in Qassim and provide a focused examination of this crucial area. This cross-sectional descriptive study aimed to characterize the demographic profiles, primary diagnoses, duration of palliative care, health-related quality of life (as measured by the EQ-5D-5L), and self-reported experiences of oncology palliative care patients in Qassim, Saudi Arabia. By providing a detailed snapshot of this patient population, this study sought to contribute valuable baseline data that can inform the development and refinement of patient-centered palliative care services in the Qassim region and beyond.

## 2. Materials and Methods

### 2.1. Study Design and Sampling

This study used a cross-sectional descriptive design to characterize the palliative care oncology patient population in Qassim, Saudi Arabia. Data were collected between January and December 2024. The study population included all eligible patients registered in the palliative care unit registries within healthcare facilities and those receiving palliative home healthcare across the Qassim Region during the designated study period. This approach aimed to provide a comprehensive census of the accessible palliative care population, resulting in a sample size of 147 participants. Data were collected via face-to-face interviews conducted by a trained research team. The EQ-5D-5L (EuroQol Research Foundation, Rotterdam, The Netherlands) and additional structured questions pertaining to patient demographics, clinical diagnoses, palliative care duration, and specific patient experience were administered.

### 2.2. Inclusion and Exclusion Criteria

#### 2.2.1. Inclusion Criteria

1. Patients of all ages receiving oncology palliative care services within the designated study period and settings in Qassim, Saudi Arabia.
2. Patients diagnosed with a cancer-related chronic, progressive, or terminal illness, necessitating palliative care.
3. Patients able to comprehend study information and provide informed consent or have a legally authorized representative (e.g., family member, guardian) available and willing to provide proxy informed consent on their behalf.

#### 2.2.2. Exclusion Criteria

1. Patients acutely unstable or too critically ill to participate safely in the interview process as determined by the attending palliative care team.
2. Patients with severe cognitive impairment who are unable to provide direct consent, and for whom a legally authorized representative is unavailable or unwilling to provide proxy consent.
3. Patients who explicitly decline participation in the study.

### 2.3. Data Collection

The EQ-5D-5L questionnaire was administered, and demographic and clinical information (age, diagnosis, and treatment details) was collected by trained members of the medical research team through face-to-face interviews. To enhance the accuracy and ensure the validity of the responses, interviews were conducted in English by a qualified researcher who provided real-time translation and clarification of each survey item in the participant’s preferred language. This approach ensured that participants fully understood the questions and provided meaningful, informed responses.

### 2.4. Data Analysis

Data were analyzed using IBM SPSS Statistics version 28.0 (IBM Corp., Armonk, NY, USA). Descriptive statistics were employed to summarize patients’ demographic and clinical characteristics, including frequencies, percentages, means, and standard deviations, as appropriate. Given the exploratory and cross-sectional design, no inferential or regression analyses were performed; however, subgroup distributions (e.g., by gender, diagnosis, and duration of care) were descriptively examined to identify observable trends. This approach provided an overview of the dataset while maintaining focus on descriptive associations consistent with the study objectives.

### 2.5. Ethical Considerations

This cross-sectional study was approved by the Regional Research Ethics Committee of the Qassim Health Cluster (IRB: [607/46/8769]). Data were anonymized during analysis to protect confidentiality, following the guidelines of the Saudi National Committee of Bioethics.

## 3. Results

### 3.1. Demographic and Clinical Characteristics of the Study Population

The study population comprised 147 palliative care oncology patients from Qassim, Saudi Arabia. As shown in Table 1, the majority of participants were female, accounting for 64.63% (n = 95) of the cohort, while males constituted 35.37% (n = 52). The age of the patients ranged from 1 to 120 years, with a mean age of 65.01 years (SD = 14.74) and a median age of 65.00 years, indicating a predominantly older adult population. Regarding primary diagnosis, breast cancer was the most prevalent, affecting 29.25% (n = 43) of patients. This was closely followed by “General Cancer/Unspecified” diagnoses, which accounted for 27.21% (n = 40) of the total cohort. Other significant diagnoses included Liver Cancer (9.52%, n = 14), Colorectal Cancer (6.80%, n = 10), and Blood Cancer (4.76%, n = 7). A small proportion (4.76%, n = 7) of patients had non-cancer diagnoses. The duration of receiving palliative care varied, with a substantial majority of patients (74.83%, n = 110) receiving care for >six months. Shorter durations included 1–3 months (10.20%, n = 15), and both less than a month and 3–5 months each accounted for 7.48% (n = 11) of the population.

### 3.2. Health-Related Quality of Life (EQ-5D-5L)

The assessment of HRQoL using the EQ-5D-5L revealed varying levels of impairment across its five dimensions within the palliative care cohort (Table 2). Regarding Mobility, 36.05% (n = 53) reported no problems, while a significant proportion experienced some degree of difficulty: 20.41% (n = 30) had slight problems, 6.80% (n = 10) had moderate problems, 14.97% (n = 22) had severe problems, and 21.77% (n = 32) were unable to walk. In terms of Self-Care, nearly half of the patients (47.62%; n = 70) reported no problems with washing or dressing. However, 18.37% (n = 27) had slight problems, 10.88% (n = 16) had moderate problems, 10.20% (n = 15) had severe problems, and 12.93% (n = 19) were unable to wash or dress. Regarding Usual Daily Activities, 31.97% (n = 47) reported no problems, 24.49% (n = 36) had slight problems, 10.20% (n = 15) had moderate problems, 12.93% (n = 19) had severe problems, and 20.41% (n = 30) were unable to perform their usual activities.

Regarding Pain/Discomfort, the distribution of responses was notable: 27.89% (n = 41) reported no pain or discomfort, and an equal percentage (27.89%, n = 41) reported slight pain or discomfort. Moderate pain was experienced by 18.37% (n = 27) of the patients, while severe pain affected 25.85% (n = 38). None of the patients reported extreme pain or discomfort. Finally, for Anxiety and Depression, 46.94% (n = 69) indicated they were not anxious or depressed. A substantial 37.41% (n = 55) reported being slightly anxious or depressed, and 15.65% (n = 23) reported being severely anxious or depressed. None of the patients reported moderate-to-extreme anxiety or depression.

Regarding anxiety and depression, 46.94% (n = 69) reported no symptoms, 37.41% (n = 55) experienced only mild symptoms, and 15.65% (n = 23) reported severe anxiety or depression. Notably, no participants selected the “moderate” or “extreme” categories, which may reflect a polarized response pattern or cultural reporting tendencies.

This polarized response pattern may reflect cultural tendencies to underreport emotional distress, underscoring the need to further validate the EQ-5D-5L for use in Arabic palliative care contexts.

### 3.3. Patient Experience and Perception of Care

Patients’ perceptions of the palliative care team and their involvement in decision-making were assessed (Table 3). Regarding the question, “Do you feel that the care team respects your choices and preferences?”, an overwhelming majority of patients (85.71%, n = 126) reported that the care team “listens to and respects my choices and preferences.” A smaller proportion, 12.24% (n = 18), felt that the care team “sometimes respects my wishes and preferences, but not always,” while only 2.04% (n = 3) reported that the care team “does not always respect my choices or preferences.”

For the question “How are your symptoms managed?”, a significant majority of patients, 68.03% (n = 100), indicated that their “Symptoms are managed effectively with minimal discomfort.” Another 22.45% (n = 33) reported that “Symptoms are manageable but not always completely.” A small percentage of patients felt their symptoms were “Neither well nor poorly managed” (6.80%, n = 10), “Not adequately managed, causing discomfort” (2.04%, n = 3), or “Very bad: Symptoms are poorly managed, causing significant discomfort” (0.68%, n = 1).

Finally, concerning patient involvement in decision-making, we asked “Do you feel involved in decision-making?” Of the patients, 68.03% (n = 100) stated “Always: I am consistently included in decisions about my care.” Additionally, 23.13% (n = 34) reported “Sometimes: I am sometimes involved in decisions.” A smaller number of patients indicated “Never: I am not involved in decisions about my care” (4.76%, n = 7) or “Rarely: I am rarely involved in decisions” (4.08%, n = 6).

## 4. Discussion

The demographic and clinical profiles of oncology palliative care patients in Qassim revealed several key characteristics relevant to service provision in the region. While the study did not perform direct QALY computations, it used the EQ-5D-5L to generate data that could support future utility estimations. The EQ-5D-5L instrument provides health-state utility values that can be translated into QALY estimates, allowing policymakers to assess the cost–utility of palliative care interventions. Establishing this conceptual link is essential for embedding value-based decision-making within the Saudi Model of Care. By quantifying health gains in utility terms, EQ-5D-5L data can inform future economic evaluations and guide the allocation of resources toward high-impact palliative care programs. This approach aligns with the broader goal of implementing value-based frameworks in SMoC. The observed female predominance (64.63%) in the cohort aligns with global epidemiological trends, in which certain life-limiting illnesses, particularly breast cancer, are more prevalent in women [14]. This demographic skew may also reflect specific healthcare-seeking behaviors or referral patterns within the Saudi Arabian context, warranting further exploration. The mean and median age of 65 years signify that palliative care services in Qassim primarily serve the older adult population, consistent with the global rise in chronic diseases among aging demographics [3]. Although the age range included patients as young as one year, the central tendency emphasized the need for geriatric-focused palliative care. The high prevalence of breast cancer (29.25%) and unspecified malignancies aligns with global and local trends [15,16], reinforcing the critical role of palliative care in oncology provision in Qassim.

The substantial proportion (27.21%) of “General Cancer/Unspecified” diagnoses suggests a potential need for more precise diagnostic coding or a reflection of the challenges in obtaining detailed medical records in a cross-sectional study, which could impact targeted care planning. The finding that nearly three-quarters of the patients (74.83%) had been receiving palliative care for more than six months is particularly noteworthy. This extended duration indicates that palliative care is provided over a prolonged period, suggesting either early integration into the disease trajectory or a sustained need for supportive care in patients with chronic and progressive conditions [17]. This contrasts with models in which palliative care is often introduced only in the very late stages of illness and highlights the potential for long-term patient–provider relationships and ongoing support needs within the Qassim palliative care system. Collectively, these findings provide a foundational understanding of the palliative care population in Qassim, emphasizing the need for services that are gender-sensitive, age-appropriate for older adults, and robust for managing diverse cancer-related symptoms over extended periods.

Although this analysis was mainly descriptive, the data indicated certain subgroup patterns, such as a higher proportion of breast cancer cases and longer care duration among female patients. Inferential analyses between variables such as gender, diagnosis, or duration of care were intentionally not conducted, as the study aimed to establish baseline characteristics rather than statistical associations. Future studies should explore these potential relationships to better understand the factors influencing quality of life and symptom control in oncology palliative populations.

Moving beyond demographics, the EQ-5D-5L results provide a comprehensive snapshot of HRQoL challenges faced by oncology palliative care patients in Qassim. While a notable proportion of patients reported no problems in dimensions such as mobility, self-care, and daily activities, a substantial percentage of patients experiencing various degrees of impairment highlights the significant physical burden associated with life-limiting illnesses in this cohort. Specifically, the combined prevalence of severe problems or the inability to perform activities (21.77% unable to walk, 12.93% unable to perform self-care, and 20.41% unable to perform usual activities) highlights critical areas for targeted intervention. These findings are consistent with the progressive nature of conditions requiring palliative care in which functional decline is a common and distressing symptom [18]. Studies have shown that mobility and self-care limitations are strongly associated with deteriorating palliative phases and an increased symptom burden. Moreover, the EQ-5D-5L has demonstrated high feasibility and sensitivity in detecting these impairments in patients with advanced malignancies [6]. These results reinforce the need for rehabilitative and supportive strategies that address functional loss and promote autonomy even in the context of progressive disease trajectories.

The pain/discomfort dimension revealed a particularly important clinical insight: while approximately 28% of patients reported no pain, an almost equal proportion reported severe pain, with a cumulative 72% experiencing some level of pain or discomfort. This high prevalence of pain, especially severe pain, indicates that pain management remains a critical area that requires enhanced focus within palliative care services in Qassim. This aligns with international reports indicating that between 35% and 55% of patients with cancer in palliative care experience moderate-to-severe pain, especially during the advanced stages of illness [19]. Effective pain control is fundamental for improving the HRQoL in palliative populations and is often the primary driver for seeking palliative care. This distribution suggests that while some patients are well managed, a significant proportion continue to experience substantial pain, which could severely impact their overall well-being, emotional stability, and ability to engage in daily life. This highlights the need for systematic pain assessment, timely intervention, and individualized pain management strategies tailored to the Qassim population.

Although this study did not include detailed data on analgesic regimens, access to pain management resources, or interdisciplinary interventions, these aspects remain essential for interpreting pain outcomes in a clinical context. Addressing such variables in future research would allow for a more comprehensive understanding of symptom control strategies and their effectiveness, ultimately guiding targeted quality improvement and multidisciplinary care initiatives.

Furthermore, the anxiety/depression findings were clinically significant. Nearly half of the patients reported no symptoms, 37.41% experienced slight anxiety/depression, and 15.65% reported severe symptoms. The absence of “moderate” or “extreme” responses may reflect a distinct pattern of patient self-reporting, possibly influenced by cultural norms, symptom interpretation, or the episodic nature of psychological distress. Alternatively, this may suggest a polarized experience in which patients either endure mild symptoms or progress directly to severe distress. Nevertheless, the combined total of over 50% of the patients experiencing some level of anxiety or depression revealed the substantial psychological burden faced by palliative care patients. This aligns with global estimates indicating that 40–60% of patients with palliative cancer experience clinically relevant psychological distress [20].

These findings emphasize the need for integrated psychosocial and spiritual support as a core component of palliative care, alongside physical symptom management. Evidence suggests that early and sustained psychosocial interventions can significantly reduce anxiety and improve the quality of life in patients with advanced illness [21]. Cumulative data on mobility, self-care, daily activities, pain, and psychological distress collectively illustrate a patient population with diverse and complex needs, reinforcing the holistic nature of palliative care and the imperative to address both physical and psychological suffering to optimize patient-centered outcomes [22].

Beyond clinical characteristics, findings regarding patient experiences and perceptions of care were largely positive, indicating strong patient satisfaction with the key aspects of palliative care in Qassim. A high percentage of patients (85.71%) reported feeling respected by the care team, and having their choices and preferences listened to was a significant strength. This suggests that a patient-centered approach is effectively implemented in practice, promoting trust, autonomy, and a sense of dignity among patients. Such positive perceptions are consistent with the global evidence showing that respectful communication, shared decision-making, and relational care are central to the quality of palliative care [23]. These elements empower patients, validate their autonomy, and enhance their emotional well-being, particularly during sensitive end-of-life discussions.

The emphasis on dignity-conserving care aligns with international frameworks that advocate individualized, culturally sensitive support as the cornerstone of ethical palliative practice [24]. The 22.45% of patients who found their symptoms manageable but not always completely managed, along with the smaller percentages reporting inadequate or very poor management, indicated areas for continuous quality improvement, perhaps focusing on individualized pain assessment and advanced symptom control strategies for a minority of patients.

Similarly, a high proportion of patients (68.03%) reported that effective symptom management with minimal discomfort was a commendable outcome. When considered alongside the EQ-5D-5L data showing that 72% of patients experienced some level of pain/discomfort, this suggests that, while pain is prevalent, its management is largely perceived as effective by the majority of patients. This highlights the distinction between the presence of symptoms and patient satisfaction in terms of how these symptoms are addressed. Similarly, international studies have shown that patients’ perceptions of symptom control are often more closely linked to communication quality and responsiveness than to symptom severity [25]. The 22.45% of those who found their symptoms manageable but not always completely managed, along with smaller percentages reporting inadequate or very poor management, indicated areas for continuous quality improvement, particularly in individualized pain assessment and advanced symptom control strategies. Evidence supports the idea that tailored interventions, including nonpharmacological approaches, multidisciplinary input, and routine outcome monitoring, can enhance symptom relief and patient satisfaction in palliative care settings [26].

The data on patient involvement in decision-making reflected a positive trend, with 68.03% consistently included in decisions about their care. This active inclusion is crucial for shared decision-making, a cornerstone of ethical and patient-centered care that promotes the autonomy, dignity, and alignment of treatment with individual values. Shared decision-making has been shown to improve treatment adherence, patient satisfaction, and clinical outcomes, particularly in palliative care settings where decisions often involve complex trade-offs [27]. The combined 7.48% (never or rarely involved) highlighted a small but important group for whom targeted communication strategies and engagement interventions could be prioritized. Overall, these patient experience metrics suggest that the palliative care team in Qassim is largely successful in delivering compassionate, respectful, and effectively managed care that actively involves patients in their treatment journey. These positive perceptions are vital for building effective therapeutic relationships, which are central to high-quality palliative care and contribute meaningfully to improved quality of life [28].

At the system level, palliative care in Saudi Arabia continues to face structural challenges, including workforce shortages, uneven service distribution, and cultural factors surrounding end-of-life care. Addressing these barriers is vital to ensure equitable and culturally appropriate access to palliative services under the Saudi Model of Care.

## 5. Conclusions

This cross-sectional descriptive study provides a foundational characterization of oncology palliative care patients in Qassim, Saudi Arabia. It offers crucial insights into their demographic profiles, clinical diagnoses, HRQoL, and perceptions of care. The findings revealed a cohort predominantly comprising older female patients (64.63%) with a significant burden of breast cancer and a notable duration of engagement in palliative care services. Although patients reported substantial impairments across various dimensions of health-related quality of life, including mobility and daily activities, and a high prevalence of pain and anxiety/depression, this study also highlighted overwhelmingly positive patient experiences. A large majority of patients felt respected by their care team, were consistently involved in decision-making, and perceived their symptoms as being effectively managed. These results highlight the complex interplay between the objective burden of illness and the subjective experience of receiving compassionate and patient-centered care. This study fills a critical gap in the literature by providing region-specific data that can serve as a vital baseline for healthcare planners and policymakers in Qassim to further refine and enhance palliative care services and ensure they remain responsive to the unique needs and preferences of this vulnerable population. From a policy perspective, these findings underscore the importance of integrating standardized HRQoL tools, such as the EQ-5D-5L, into routine palliative care practice under the Saudi Model of Care. Such integration would facilitate continuous outcome monitoring and guide evidence-based improvements in service delivery and resource allocation.

Despite these valuable insights, this study had several limitations. Its cross-sectional design precludes the establishment of cause-and-effect relationships or assessment of changes in patient status over time. The reliance on self-reported data for quality of life and patient experience introduces a potential for recall bias. Additionally, social desirability bias may have influenced patient responses, as participants could have tended to overreport positive experiences due to cultural or interpersonal factors common in palliative care contexts. Furthermore, this study was conducted in a single region of Saudi Arabia, which limits the generalizability of the findings to a broader national context or other cultural settings. The absence of comparative groups (e.g., non-oncology palliative populations or cohorts from other regions) further constrains external validity and contextual interpretation. The sample size, although adequate for descriptive purposes, may have restricted our ability to perform robust subgroup analyses. Future research should build upon these descriptive findings to explore associations between patient characteristics and outcomes and evaluate the impact of specific interventions aimed at improving the quality of life and care experiences.

## Figures and Tables

**Table 1:** Socio-demographic and clinical characteristics of the study population (N = 147).

*Characteristic*	*Category*	*Count (* *n* *)*	*Percent (%)*
*Sex*	Female	95	64.63
	Male	52	35.37
*Age (Years)*	Mean (SD)	65.01 (14.74)	
	Median	65.00	
	Minimum	1	
	Maximum	120	
*Primary Diagnosis*	Breast Cancer	43	29.25
	General Cancer/Unspecified	40	27.21
	Liver Cancer	14	9.52
	Colorectal Cancer	10	6.80
	Blood Cancer	7	4.76
	Non-Cancer Diagnosis	7	4.76
	Uterine Cancer	7	4.76
	Prostate Cancer	6	4.08
	Advanced Cancer (Metastatic)	4	2.72
	Bone Cancer	3	2.04
	Brain Tumor	3	2.04
	Pancreatic Cancer	2	1.36
	Lung Cancer	1	0.68
*Duration of Care*	Less than a month	11	7.48
	1–3 months	15	10.20
	3–5 months	11	7.48
	More than 6 months	110	74.83

**Table 2:** Health-related quality of life profile (EQ-5D-5L) of palliative care patients (N = 147).

*Dimension*	*Response Category*	*Count (* *n* *)*	*Percent (%)*
*Mobility*	I have no problems in walking about	53	36.05
	I have slight problems in walking about	30	20.41
	I have moderate problems in walking about	10	6.80
	I have severe problems in walking about	22	14.97
	I am unable to walk about	32	21.77
*Self-Care*	I have no problems washing or dressing myself	70	47.62
	I have slight problems washing or dressing myself	27	18.37
	I have moderate problems washing or dressing myself	16	10.88
	I have severe problems washing or dressing myself	15	10.20
	I am unable to wash or dress myself	19	12.93
*Daily Activities*	I have no problems doing my usual activities	47	31.97
	I have slight problems doing my usual activities	36	24.49
	I have moderate problems doing my usual activities	15	10.20
	I have severe problems doing my usual activities	19	12.93
	I am unable to do my usual activities	30	20.41
*Pain/Discomfort*	I have no pain or discomfort	41	27.89
	I have slight pain or discomfort	41	27.89
	I have moderate pain or discomfort	27	18.37
	I have severe pain or discomfort	38	25.85
	I have extreme pain or discomfort	0	0.00
*Anxiety/Depression*	I am not anxious or depressed	69	46.94
	I am slightly anxious or depressed	55	37.41
	I am moderately anxious or depressed	0	0.00
	I am severely anxious or depressed	23	15.65
	I am extremely anxious or depressed	0	0.00

**Table 3:** Patient perceptions of care and involvement in decision-making (N = 147).

*Question*	*Response Category*	*Count (n)*	*Percent (%)*
*Do you feel that the care team respects your choices and preferences?*	Yes: The care team listens to and respects my choices and preferences.	126	85.71
	Somewhat: The care team sometimes respects my wishes and preferences, but not always.	18	12.24
	No: The care team does not always respect my choices or preferences.	3	2.04
*How are your symptoms managed?*	Very good: Symptoms are managed effectively, with minimal discomfort.	100	68.03
	Fairly good: Symptoms are manageable but not always completely.	33	22.45
	Neutral: Symptoms are neither well nor poorly managed.	10	6.80
	Bad: Symptoms are not adequately managed, causing discomfort.	3	2.04
	Very bad: Symptoms are poorly managed, causing significant discomfort.	1	0.68
*Do you feel involved in decision-making?*	Always: I am consistently included in decisions about my care.	100	68.03
	Sometimes: I am sometimes involved in decisions.	34	23.13
	Never: I am not involved in decisions about my care.	7	4.76
	Rarely: I am rarely involved in decisions.	6	4.08

## Data Availability

Data available on request from the corresponding author. Data derived from MOH data and cannot be found in public databases or published publicly. Restrictions apply to the database, but it may be possible to share if approved by MOH.

## Abbreviations

The following abbreviations are used in this manuscript:

SMoC	Saudi Model of Care
QALY	Quality-Adjusted Life Year
WHO	World Health Organization
HRQoL	Health-Related Quality of Life
EQ-5D-5L	EuroQol Five-Dimension Five-Level Questionnaire
